# Supplementary material for: Integrative pan-resistome and transcriptomic characterization reveals differential gene expression signatures in carbapenem-resistant Acinetobacter baumannii: Insights into efflux pump regulation and therapeutic targeting strategies
Source: PLoS One. 2026 Jan 16;21(1):e0340266. doi: 10.1371/journal.pone.0340266 (PMC12810843; doi:10.1371/journal.pone.0340266)
Supplement: Attachment — Submitted filename: Lamisa_review.docx [file pone.0340266.s002.docx]

This study investigates the pangenome, resistome, and transcriptomic responses of *Acinetobacter baumannii* to sub-MIC antibiotic exposure, with a focus on efflux pump-mediated resistance. It also evaluates potential marine-derived inhibitors of the MacB efflux transporter through in silico screening and molecular dynamics simulations. While the study addresses an important question in antimicrobial resistance and employs a comprehensive, multi-layered analytical approach, several aspects of the methodology and data presentation would benefit from clarification and additional detail. My specific comments and suggestions are outlined below:

1. The authors describe a standard RNA-seq workflow for multiple *A. baumannii* strains, with experiments performed in duplicate as indicated in Table 1. While the overall approach, including quality assessment with FastQC, trimming with Trimmomatic, alignment with Subread, and quantification with featureCounts, is appropriate, there are several points that require clarification. The total sample size per condition should be explicitly stated in the text to ensure transparency. Furthermore, there is a discrepancy between the methodology and Figure 1, where HISAT2 and DESeq2 are indicated, whereas the Methods section describes Subread-align, featureCounts, and edgeR, which may cause confusion. To improve reproducibility, the authors should also provide details on quality filtering parameters, software versions, annotation release, and replicate design.

2. The use of PubMLST for Multi-Locus Sequence Typing and Roary for pan-genome analysis is appropriate, and Phandango visualization provides an effective means to explore gene presence and absence patterns. However, the section would benefit from additional details regarding the genomes analyzed, including accession numbers, source information, and assembly quality. While the thresholds for defining core genes are specified (>95% amino acid identity and >99% presence), other parameters used in Roary should be clarified. Similarly, more information on how Phandango was used for visualization would enhance clarity and reproducibility.

3. The authors appropriately applied Abricate with the CARD and VirulenceFinder databases to identify antimicrobial resistance and virulence-associated genes. To ensure reproducibility, it would be helpful to specify the exact database versions used, provide precise criteria or parameters for calling gene presence, and include accession numbers or sources for the genome assemblies analyzed. These additions would allow readers to better interpret the results and replicate the analysis.

4. The authors appropriately used edgeR for differential expression analysis, including normalization with the TMM method and statistical testing via the quasi-likelihood framework. However, the number of biological replicates per condition should be explicitly stated, as this is essential for assessing statistical power and reliability. There is a minor but important point regarding the reported thresholds: the manuscript mentions (log_2_FC) rather than |log_2_FC|. Using the absolute value is important when applying a fold-change cutoff irrespective of direction, and the authors should clarify whether up- and down-regulated genes were treated separately or together. Additionally, the authors should indicate whether batch effects were assessed or corrected.
